# Surgical and embryological perspective of a big loop of internal carotid artery extending laterally beyond internal jugular vein

**DOI:** 10.1007/s00276-020-02619-z

**Published:** 2020-11-24

**Authors:** Satheesha B. Nayak, Surekha D. Shetty

**Affiliations:** Department of Anatomy, Melaka Manipal Medical College (Manipal Campus), Manipal Academy of Higher Education (MAHE), Madhav Nagar, Manipal, Karnataka 576104 India

**Keywords:** Variation, Internal carotid artery, Loop, Internal jugular vein, Vascular surgery

## Abstract

Knowledge of variations of the internal carotid artery is significant to surgeons and radiologists. The internal carotid artery normally runs a straight course in the neck. Its anomalies can lead to its iatrogenic injuries. We report a case of a large loop of the internal carotid artery in a male cadaver aged about 75 years. The common carotid artery terminated by dividing it into the external carotid artery and internal carotid arteries at the level of the upper border of the thyroid cartilage. From the level of origin, the internal carotid artery coursed upwards, backwards and laterally, and formed a large loop behind the internal jugular vein. The variation was found on the left side of the neck and was unilateral. The uncommon looping of the internal carotid artery might result in altered blood flow to the brain and may lead to misperceptions in surgical, imaging, and invasive procedures.

## Introduction

The internal carotid artery (ICA) is the larger terminal branch of the common carotid artery (CCA), originates at the level of the upper border of the lamina of the thyroid cartilage. It ascends vertically in the carotid sheath and at the base of the skull enters the carotid canal. It passes through the cavernous sinus in the cranial cavity and later divides into anterior and middle cerebral arteries at the base of the brain. The ICA is the major artery of the brain, eye, and internal ear [27]. The ICA has a straight course in the neck. It can be straight, curved, or angled [2].

The ICA is the principal source of blood supply to the brain. Its reported variations include coiling, looping, kinking or tortuosities, which may cause significant neurovascular problems due to changes in the dynamics of blood flow. The tortuous course of the ICA in the neck has a high chance of being injured during head and neck surgeries. Tortuosity, kinking, and looping of the internal carotid artery has been well recognized for many years. Anatomists have described this artery as being remarkable for the number of curvatures in its course. Looping of ICA was mentioned as pulsatile swelling in the neck by Coulson [8]. Otolaryngologists have been concerned about looped ICA, because it can be damaged during tonsillectomy or adenoidectomy may lead to fatal haemorrhage [12].

Any anatomical variations of the carotid arteries could be secondary to embryological defects [24]. S or C-shaped elongations and tortuosities, kinkings, and loops of the ICA are thought to be congenital anomalies [6]. Their genesis can be explained in terms of the embryological development of the branchial arch arteries. These variations are of great importance in clinical diagnoses, during neck surgeries & while carrying out carotid angiography. We report a significant curve in the course of the internal carotid artery in the neck which could be of clinical importance. We discuss its clinical and embryological significance.

## Case report

**Fig. 1:**
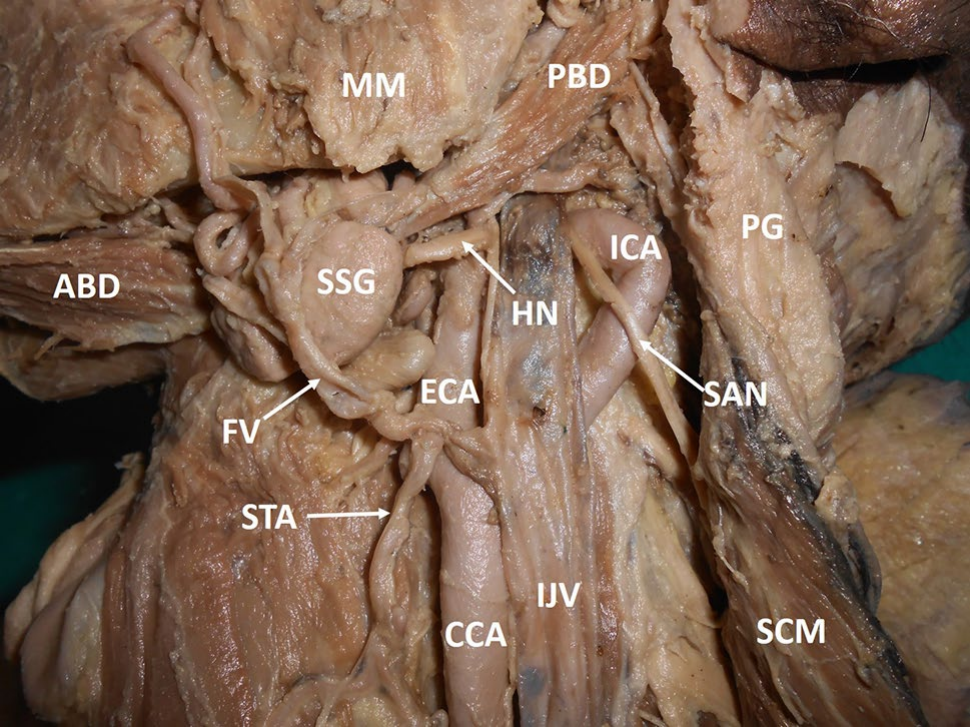
Dissection of the left carotid triangle (lateral view) showing the loop of the internal carotid artery. *CCA* common carotid artery, *ECA* external carotid artery, *ICA* internal carotid artery, *IJV* internal jugular vein, *SCM* sternocleidomastoid, *SSG* submandibular salivary gland, PBD posterior belly of digastric, *ABD* anterior belly of digastric, *FV* facial vein, *STA* superior thyroid artery, *MM* masseter muscle, *SAN* spinal accessory nerve, *HN* hypoglossal nerve, *PG* parotid gland

**Fig. 2:**
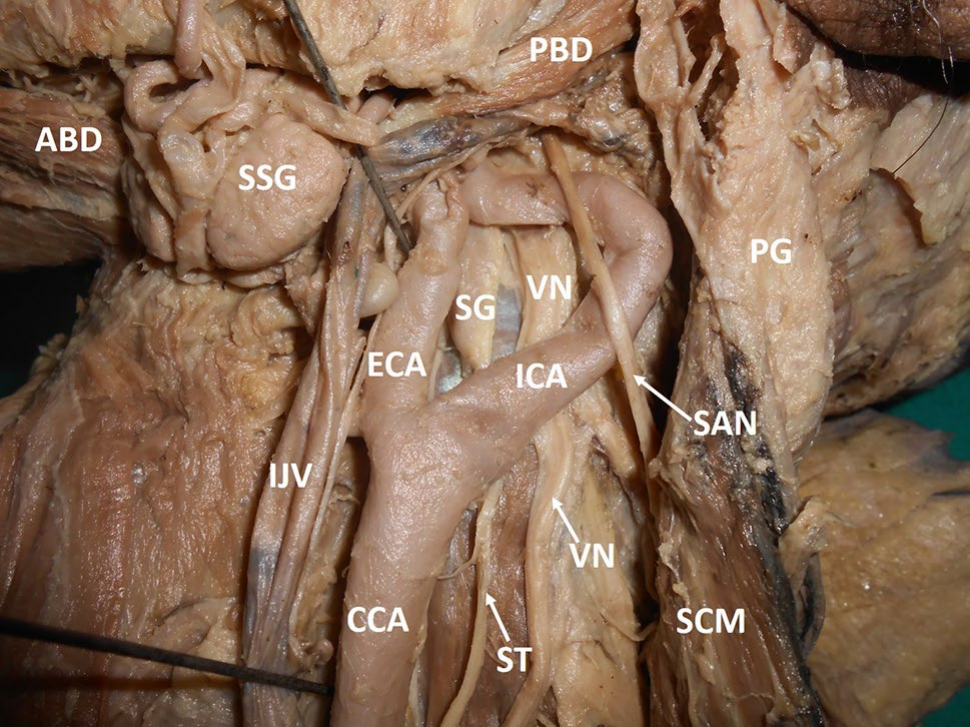
Dissection of the left carotid triangle (lateral view) showing the loop of the internal carotid artery. Internal jugular vein has been pulled medially. *CCA* common carotid artery, *ECA* external carotid artery, *ICA* internal carotid artery, *IJV* internal jugular vein, *SCM* sternocleidomastoid, *SSG* submandibular salivary gland, *PBD* posterior belly of digastric, *ABD* anterior belly of digastric, *SAN* spinal accessory nerve, *VN* vagus nerve, *SG* superior cervical sympathetic ganglion, *ST* sympathetic trunk, *PG* parotid gland

During Routine dissection classes for undergraduate medical students, a large loop of the internal carotid artery was noted in a male cadaver aged about 75 years. The variation was found on the left side of the neck and was unilateral. The CCA, terminated by dividing into an external carotid artery (ECA) and ICA at the level of the upper border of the thyroid cartilage. From the level of origin, the ICA coursed upwards, backwards and laterally, and formed a large loop behind the internal jugular vein (IJV). The part of the artery that formed the loop, measured about 7 cm and the loop projected beyond the lateral border of the IJV in the carotid sheath (Fig. 1). After forming the loop, the artery coursed medially, passed behind the ECA and resumed its normal course until the base of the skull. The vagus nerve and the superior cervical sympathetic ganglion were situated in the concavity of the loop, between ECA and ICA and were overlapped by the IJV (Fig. 2). The spinal accessory nerve passed superficial to the loop, between the IJV and the ICA (Fig. 2). The sternocleidomastoid muscle overlapped the loop of ICA.

## Discussion

Usually, from its origin, till the skull base, ICA follows a straight course. However, there are reports on the formation of loops in the cervical region. About 10–40% of humans might have aberrations in the course of the ICA. These may be congenital or secondary to advancing age or atherosclerosis [20]. Frequent abnormalities of ICA include tortuosity, kinking, and coiling [10]. And most of them are found during carotid angiography [15].

Often, loops of ICA could be mistaken for a tumour or an abscess during routine ENT procedures [29]. Abnormal loops might produce obstructive sleep apnea due to the compression of the pharyngeal wall in recumbent posture [21]. Aberrant ICA segments may also cause difficulty in swallowing and speech or a sensation of a foreign body in the pharynx. Oropharyngeal pulsatile mass, longstanding hoarseness, foreign body sensation, and upper respiratory distress is also seen in most of the patients with an aberrant ICA [16]. Paulsen et al. [22] have discussed tortuous ICA and its clinical significance in tonsil and pharynx. Before surgery of adenoids, tonsil, and pharyngeal space, the patient has to be checked for ICA and its variations to prevent severe hemorrhage [10, 20].

The unusual looping of ICA at its beginning might result in altered blood flow to the brain [17]. Looping of the artery in the lateral pharyngeal space has also been noted by Tillmann and Christofides [28]. Nayak and Kumar [18] have reported a unilateral occurrence of multiple loops of ECA and ICA on the left side of the neck. In another study, both the ECA and ICA were found to have anomalously curved S-shaped courses in a 70-year-old female cadaver on the right side of the neck [26].

Kinking occurs mostly in elderly men and coiling is more common in younger women [23]. Kink is common in arteriosclerosis and hypertensive persons [19]. The kinks and coils are congenital in children and younger patients [4]. Among the variations of the ICA, an unusual retropharyngeal course can mimic parapharyngeal neoplasm and may increase the risk of vascular injury during the pharyngeal intervention [1]. The ICA may have a tortuous course with a prominent curve or a horseshoe shape or may form a complete loop in the upper part of the neck. This kinking of the artery may be due to cardiovascular disease [5]. Looping or tortuosity of ICA can also be observed with advancing age [11].

Carotid artery anomalies (agenesis, aplasia, and hypoplasia) may be related to their development or their course (coiling, kinking, and tortuosity). Carotid arteries rarely develop in uncommon anatomical spaces, extending to the retropharyngeal or prevertebral regions [9]. The ICA develops on both sides from the third aortic arch artery (third branchial arch artery) and the cranial part of the dorsal aorta. Tortuosity of carotid arteries is common during foetal life and in infants. Usually, the descent of the large blood vessels and heart into the mediastinal space during incessant development leads to elongation and straightening of the artery [13, 24]. Loop may persist if there is a failure of process, incomplete development, or accelerated linear growth of the artery [1, 25]. Coiling and complete loop formation of the ICA is observed in 5–15% of patients during angiographic procedures. This appearance is supposed to be partly developmental, unrelated to either age or hypertension [3].

Variations in the course of the ICA may cause alteration in blood flow dynamics leading to numerous neurological manifestations [30]. Carsuzaaa et al. [7] reported the case of the death of a 7-year-old child consecutively to cardiac arrest during the surgery of cholesteatoma. In an autopsy, the right carotid loop and left carotid kink were found which had led to cerebral hypoperfusion. The loops of ICA might even remain silent, without causing any harm to the person carrying them. A case of an asymptomatic patient with a bilateral atypical course of ICAs, has been found incidentally during a physical examination [31]. Cerebral emboli and vessel occlusion are common because of the tortuosity of the ICA which causes cerebral ischemia [14].

The internal carotid artery is closely related to the deep cervical lymph nodes. During the removal of cervical lymph nodes, the surgeon should be able to differentiate the resemblance of the loop to a node and the presence of node in the loop. The artery is particularly dangerous when it is in contact with the tonsillar fossa. It is important to know the anatomy of vessels and variations for interpretation of diagnostic and interventional vascular procedures during surgeries to prevent complications.

## How is the current case different than the previously reported cases?

The current case is unique compared to the previously reported cases due to its lateral direction. Most of the previously reported vessels extend dorsomedially into the retropharyngeal space causing symptoms related to the pharynx and larynx. In the current case, the loop of the ICA projected well beyond the IJV, laterally deep to sternocleidomastoid. This placement could be surgically significant because of its rarity. During surgical removal of the deep cervical lymph nodes, there is a high chance for the loop to be injured. Another difference between the current case and the previously published reports is that the loop was closely related to the superior cervical sympathetic ganglion and the spinal accessory nerve. The loop might put pressure on both of them causing Horner’s syndrome or a wry neck. The current looping of the artery was unilateral. Hence, it would have happened in post-natal life. Usually, congenital looping of the artery happens if the heart fails to shift to the mediastinum and in such cases, arteries of both sides should present loops. The current case could be of importance to radiologists, head and neck surgeons, and neurologists.
